# Turf Quality and Physiological Responses to Summer Stress in Four Creeping Bentgrass Cultivars in a Subtropical Zone

**DOI:** 10.3390/plants11050665

**Published:** 2022-02-28

**Authors:** Zhou Li, Weihang Zeng, Bizhen Cheng, Jie Xu, Liebao Han, Yan Peng

**Affiliations:** 1 Department of Turf Science and Engineering, College of Grassland Science and Technology, Sichuan Agricultural University, Chengdu 611130, China; lizhou1986814@163.com (Z.L.); zengwh0123@163.com (W.Z.); chengbizhengrass@163.com (B.C.); 2 Institute of Turfgrass Science, Beijing Forestry University, Beijing 100083, China; 3 Chengdu Times Green Spaces Horticulture Co., Ltd., Chengdu 610300, China; xujie8231881@163.com

**Keywords:** high temperature, oxidative damage, photosynthesis, osmotic adjustment, photochemical efficiency

## Abstract

Cool-season creeping bentgrass (*Agrostis stolonifera*) has the ability to form fine sports turf, but high temperatures result in summer bentgrass decline (SBD), especially in transitional and subtropical zones. Physiological responses in combination with the alteration in turf quality (TQ) will contribute to a better understanding of SBD in a subtropical zone. Field experiments were conducted from 2017 to 2019 to test the adaptability to summer stress among four cultivars (13M, Penncross, Seaside II, and PA-1). A constant ambient high temperature above 30 °C significantly decreased the TQ of the four cultivars during the summer months in 2017, 2018, and 2019. Significant declines in the chlorophyll content, photochemical efficiency of photosystem II (Fv/Fm and PIABS), leaf relative water content (RWC), and osmotic potential (OP) were induced by summer stress, whereas gradual increases in water-soluble carbohydrates, proline, hydrogen peroxide (H_2_O_2_), malondialdehyde (MDA), and electrolyte leakage (EL) were observed in the four cultivars during the summer months. The 13M and Penncross cultivars exhibited better performance than Seaside II and PA-1 in response to summer stress from 2017 to 2019, which is associated with better maintenance of photosynthesis, water status, WSC and proline accumulation, and cell membrane stability. The 13M and Penncross cultivars could be used as potential candidates for turf establishment in a subtropical zone. Physiological responses together with alterations in TQ also provided critical information for the breeding and development of germplasm with heat tolerance in creeping bentgrass species.

## 1. Introduction

Cool-season turfgrasses such as creeping bentgrass (*Agrostis stolonifera*), perennial ryegrass (*Lolium perenne*), and Kentucky bluegrass (*Poa pratensis*) have an optimal range of growth temperature from 15 to 24 °C and are highly susceptible to sustained high temperatures in the summer [1]. Creeping bentgrass, characterized by rapid thatch accumulation, fine texture, and low mowing height, is widely used in sports turfs such as golf green and tennis lawns. In transitional and subtropical zones, the high temperature during the summer months is a critical stress factor resulting in summer bentgrass decline (SBD) [2,3]. Maintenance practices including water and fertilizer management, the alteration of mowing height, and the application of plant growth regulators have been utilized to alleviate SBD [3,4,5,6]. In addition to managerial actions, the identification and selection of creeping bentgrass cultivars that adapt to one particular climate type are of primary importance to reduce maintenance cost because environmental adaptability to heat stress varies with creeping bentgrass cultivars or genotypes [7,8]. Previous studies have identified some heat-tolerant creeping bentgrass cultivars such as ‘L-93’ in controlled growth chambers or under field conditions [2,9]. The field performance of different creeping bentgrass cultivars, especially for those newly developed or promoted cultivars, should be further assessed during the summer months in different climatic regions.

Various metabolic processes in plants are interrupted by heat stress. For example, high temperatures limit carbohydrate production and supply for growth maintenance in cool-season turfgrass, mainly due to accelerated respiration and reduced photosynthesis [10]. Carbohydrates and other osmolytes such as proline (Pro) exhibit a variety of roles in osmotic adjustment, antioxidant, and metabolic homeostasis [11]. It has been suggested that carbohydrate production is beneficial in alleviating SBD [2]. Proline accumulation and metabolism are also associated with better adaptation to enhanced heat tolerance in creeping bentgrass [12,13,14]. In addition, the heat-induced decline in the turf quality (TQ) of creeping bentgrass is related to accelerated leaf senescence, as demonstrated by the chlorosis of turf [15]. One of the core factors responsible for heat-induced leaf senescence in bentgrass species is reactive oxygen species (ROS) overaccumulation leading to membrane peroxidation [16,17,18,19]. However, most of the controlled studies in growth chambers limit a deep understanding of SBD in relation to the multiple physiological responses of different creeping bentgrass genotypes in a subtropical zone.

As one of the most commonly used cool-season turfgrasses in golf greens, the heat-induced decline in the TQ of creeping bentgrass is the most intractable problem to turf managers during hot summers. The objectives of this study were to evaluate the summer performance of four different creeping bentgrass cultivars (‘Penncross’, ‘13M’, ‘Seaside II’, and ‘PA-1’) and to further examine common or different physiological responses, including leaf water relation, photosynthetic performance, osmolytes accumulation, and oxidative damage associated with heat tolerance among these cultivars during the summer months under field conditions. Physiological responses in combination with the alteration in TQ will contribute to a better understanding of SBD in a subtropical zone.

## 2. Materials and Methods

### 2.1. Plant Materials and Treatments

**Figure 1:**
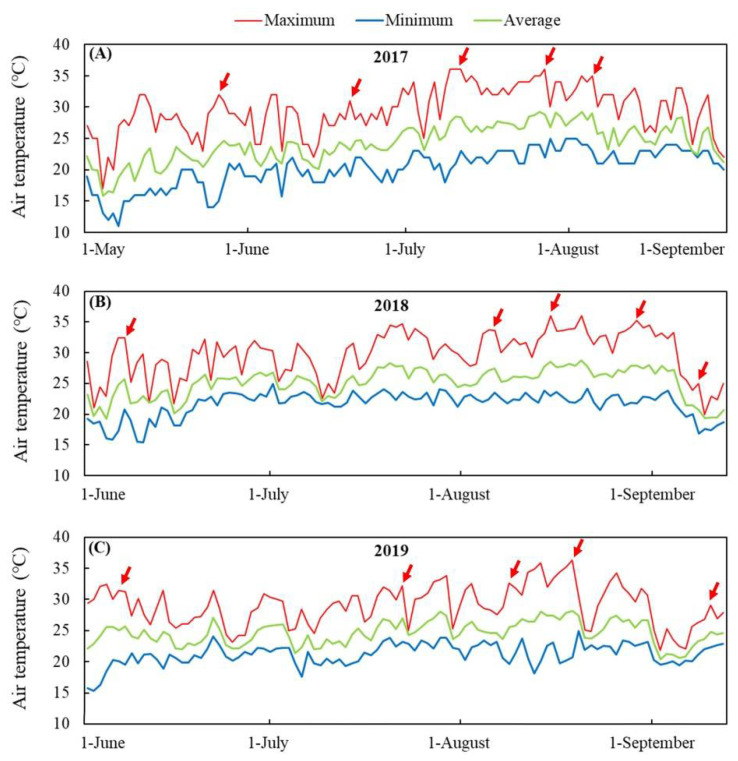
Daily maximum, minimum, and average air temperature in (**A**) 2017, (**B**) 2018, and (**C**) 2019 in the Research Farm of Sichuan Agricultural University (Chongzhou, Sichuan, China, east longitude 103°07′–103°49′ and north latitude 30°30′–30°53′). Red arrows indicate sampling dates.

The experiment was conducted in the Research Farm of Sichuan Agricultural University which is located in southwest China (Chongzhou, Sichuan, east longitude 103°07′-103°49′ and north latitude 30°30′–30°53′). The area has a typical subtropical-monsoon climate with an annual mean temperature between 16 °C and 17 °C. Reclaimed soils (loams: sands, 2:1) were used as the plant layer with the supply of 2.0 g m^−2^ fertilizers (nitrogen: phosphorus: potassium, 3:1:1). The seeds of four different creeping bentgrass cultivars (Penncross, 13M, Seaside II, or PA-1) were sown evenly in each 2 m × 2 m plot in October 2016. The seeding rate was 10 g m^−2^ for each cultivar. After nearly 8 months of establishment from October 2016 to May 2017, the turfgrass coverage of all cultivars reached nearly 100%, and mowing height was maintained at 2 cm. The summer tolerance of the four cultivars was evaluated from May to August 2017, June to September 2018, and June to September 2019. During summer stress, all turfs were irrigated daily with city water to avoid drought stress. The irrigation time changed within the year of the experiment in relation to seasonal temperatures and soil conditions. The maximum, minimum, and average daily air temperatures are demonstrated in Figure 1A–C. The total number of days where the maximum air temperatures were above 30 °C were 64, 59, and 45 days in the summers of 2017, 2018, and 2019, respectively (Figure 1A–C), which indicated that all materials suffered from a long period of high temperature stress in the summers of 2017, 2018, and 2019. Dead spots in each turf plot were replaced by new sods in the spring to make all turfs uniform before the summer stress of the next year.

### 2.2. Measurements of Turf Quality and Photosynthetic Parameters

TQ was evaluated based on presentation quality, which included uniformity, color, and density, and was rated a 1, 6, or 9 if the presentation quality of the turf was the worst (brown and desiccated), minimally acceptable (pale green and a 10% decline in denseness), or excellent (green and dense), respectively [20]. For the Chl content, fresh leaves (0.2 g) were collected from each turf and soaked in a 20 mL of 80% acetone and 95% ethanol (1:1, *v*:*v*) solution for 48 h in the dark. The absorbance of the extraction liquid was detected at 645 and 663 nm with a spectrophotometer (Spectronic Instruments, Rochester, NY, USA) [21]. A portable Chl fluorescence system (Pocket PEA, Hansatech, Norfolk, UK) was used to determine the photochemical efficiency (Fv/Fm) and the performance index on absorption basis (PIABS). In brief, a single layer of leaves was covered by leaf clips for 30 min for dark adaptation, and then, the Fv/Fm and PIABS data were recorded. The net photosynthetic rate (Pn), transpiration rate, and instantaneous water use efficiency (WUE) were detected by using a portable photosynthetic system (CIRAS-3, PP Systems, Amesbury, MA, USA) that provided stable and continuous light and carbon dioxide conditions (400 μL/L CO_2_ and 800 μmol photon m^−2^ red and blue lights).

### 2.3. Measurements of Water Status and Osmolyte Contents

The water status in leaves was evaluated with the leaf relative water content (RWC) and the osmotic potential (OP). Fresh leaves were collected from the turf and were weighed immediately to obtain the fresh weight (FW), and then, these leaves were soaked in distilled water for 12 h to weigh turgid weight (TW). Dry weight (DW) was obtained after leaves were dried in an oven at 105 °C for 2 h and then at 80 °C for 72 h. The leaf RWC (%) was calculated based on the formula (RWC (%) = [(FW − DW)/(TW − DW)] × 100) [22]. Osmotic potential (OP) was determined by using an osmometer 4420 (Wescor, Inc., Logan, UT, USA), and the assay method has been recorded in detail in the studies of [23,24]. For the analysis of the proline content, fresh leaves (0.2 g) were extracted in boiling water for 20 min in a 10 mL mixture containing 3% sulfosalicylic acid, and then, 2 mL of glacial acetic acid and 3 mL of 2.5% ninhydrine were added. The reaction mixture was boiled for 30 min. The 5 mL of toluene was added and shaken up. The absorbance of upper toluene was recorded at 520 nm using a Genesys 2PC spectrophotometer (Spectronic Instruments, Rochester, NY, USA) [25]. Water-soluble carbohydrate (WSC) levels were determined according to the method of [26] with some modification, which has been clearly demonstrated in our previous study [27].

### 2.4. Measurements of Oxidative Damage and Membrane Stability

The hydrogen peroxide (H_2_O_2_) content was detected by using 0.2 g of fresh leaves which were homogenized in 10 mL of 0.1% TCA. After being centrifuged at 10,000× *g* for 15 min, 0.5 mL of the supernatant was mixed with 0.5 mL of 10 mM potassium phosphate and 1 mL of 1 M potassium iodide and then placed in the dark for 5 min. The absorbance of the reaction mixture was read at 390 nm [28]. For the analysis of malondialdehyde (MDA) content, 3 mL of 50 mM cold phosphate buffer (pH 7.8) was used to extract 0.2 g of fresh leaves. The supernatant was obtained after the homogenate was centrifuged at 10,000× *g* for 30 min at 4 °C, and then, 0.5 mL of the supernatant was mixed with 1 mL of the reaction solution (20% *w*/*v* trichloroacetic acid and 0.5% *w*/*v* thiobarbituric acid). After being heated in a boiling water bath for 15 min, the reaction mixture was centrifuged at 8000× *g* for 10 min. The absorbance of 1.5 mL of supernatant was measured at 532 and 600 nm [29]. Leaf electrolyte leakage (EL) was calculated based on the formula (%) = C_initial_/C_max_ × 100, where C_initial_ indicated the initial conductivity and C_max_ presented the max conductivity. Fresh leaves (0.2 g) were soaked instantly in 50 mL of distilled water for 24 h to detect the C_initial_. These samples were autoclaved at 120 °C for 20 min and cooled down to room temperature to detect the C_max_ using a conductivity meter (YSI Model 32, Yellow Spring, OH, USA) [30].

### 2.5. Experimental Design and Statistical Analysis

The experimental design was a randomized blocks design, and each treatment (each cultivar) was replicated four times (four test plots) in the field. All measurements were sampled in each plot including five subsamples, and the average value of the five subsamples was regarded as the effective value in each plot. Variations among the four cultivars in response to summer stress were analyzed by the general linear model procedure of Statistical Product and Service Solutions 24 (SPSS Institute, IBM, Armonk, NY, USA, 2018). Differences among treatments (cultivars) were determined by using the least significant difference (LSD) at *p* ≤ 0.05.

## 3. Results

### 3.1. Turf Quality and Photosynthetic Parameters Affected by Summer Stress

**Figure 2:**
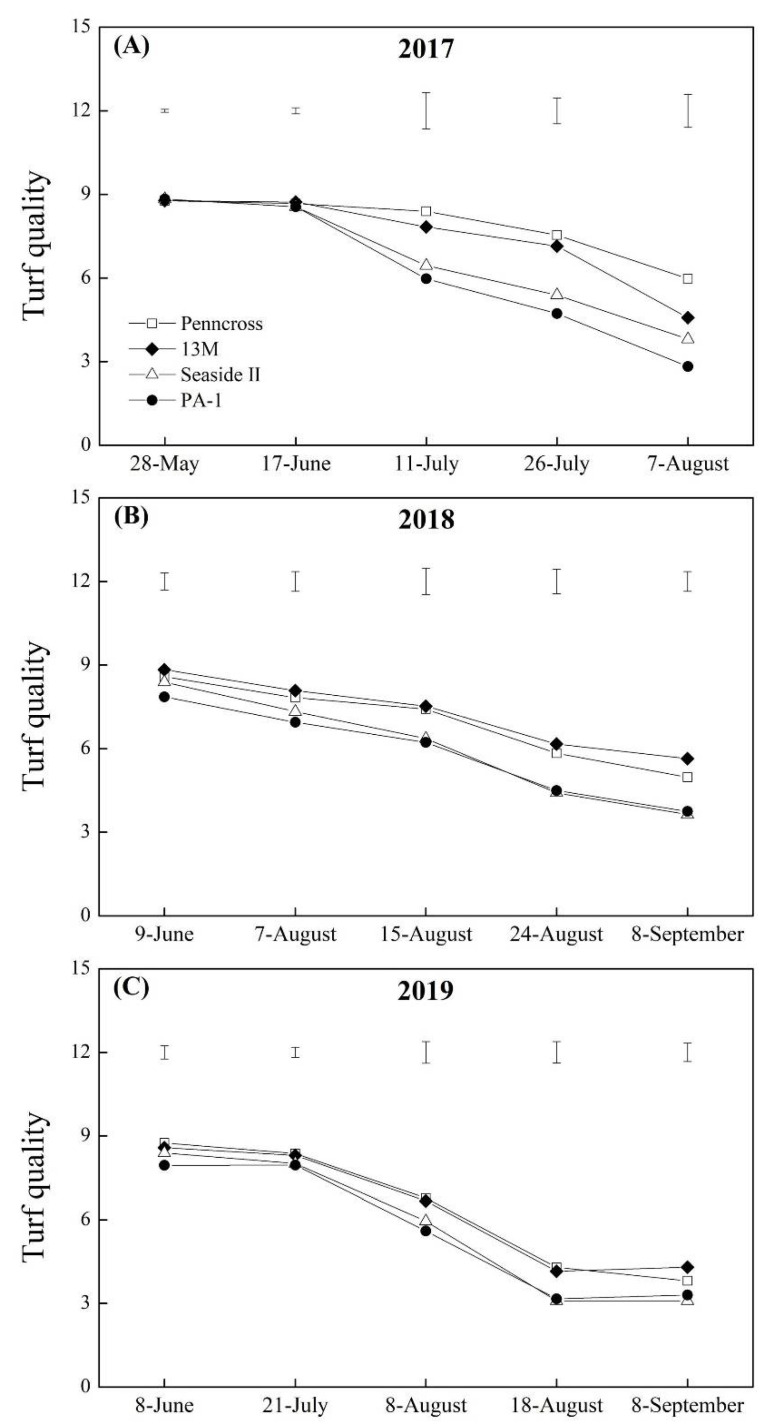
Change in turf quality (TQ) of four different creeping bentgrass cultivars (Penncross, 13M, Seaside II, and PA-1) during summer in (**A**) 2017, (**B**) 2018, and (**C**) 2019. Vertical bars above curves indicate least significant difference (LSD) values (*p* < 0.05) at a given day.

**Figure 3:**
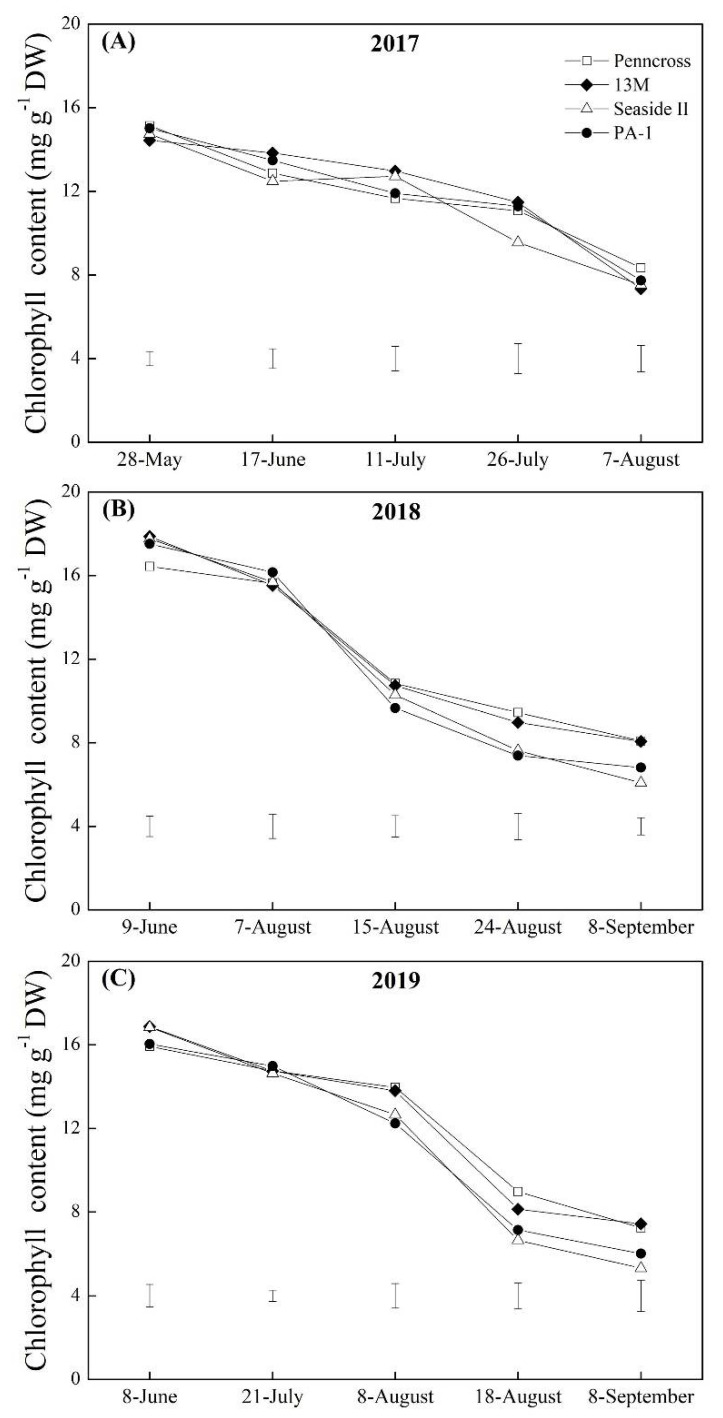
Change in chlorophyll (Chl) content of four different creeping bentgrass cultivars (Penncross, 13M, Seaside II, and PA-1) during summer in (**A**) 2017, (**B**) 2018, and (**C**) 2019. Vertical bars below curves indicate least significant difference (LSD) values (*p* < 0.05) at a given day.

The TQ of the four cultivars declined gradually during the summer months in 2017, 2018, and 2019, but Penncross and 13M showed higher TQ than Seaside II and PA-1 in response to summer stress (Figure 2A–C). On 7 August 2017, the highest TQ was observed in the Penncross cultivar (Figure 2A); however, the 13M cultivar exhibited the highest TQ out of the four cultivars on 8 September 2018 and 2019 (Figure 2B,C). The Chl content in all cultivars decreased with the development of summer stress from June to September in 2017, 2018, and 2019 (Figure 3A–C). The Penncross, 13M, and PA-1 cultivars had a significantly higher TQ than Seaside II on July 26th, whereas no significant difference in the Chl content among the four cultivars was detected on other sampling dates during the summer in 2017 (Figure 3A). On 8 September 2018 and 2019, significantly lower Chl content was observed in PA-1 and Seaside II as compared to that in Penncross and 13M (Figure 3B,C).

**Figure 4:**
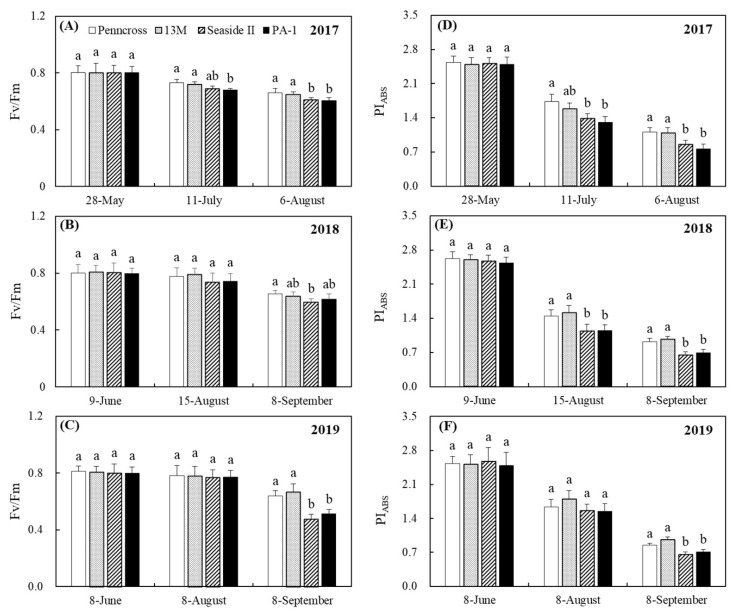
Change in (**A**–**C**) photochemical efficiency (Fv/Fm) and (**D**–**F**) performance index on absorption basis (PIABS) of four different creeping bentgrass cultivars (Penncross, 13M, Seaside II, and PA-1) during summer in (**A**) 2017, (**B**) 2018, and (**C**) 2019. Vertical bars indicate ±SE of mean (n = 4). Different letters indicate significant differences (*p* < 0.05) at a given day.

**Figure 5:**
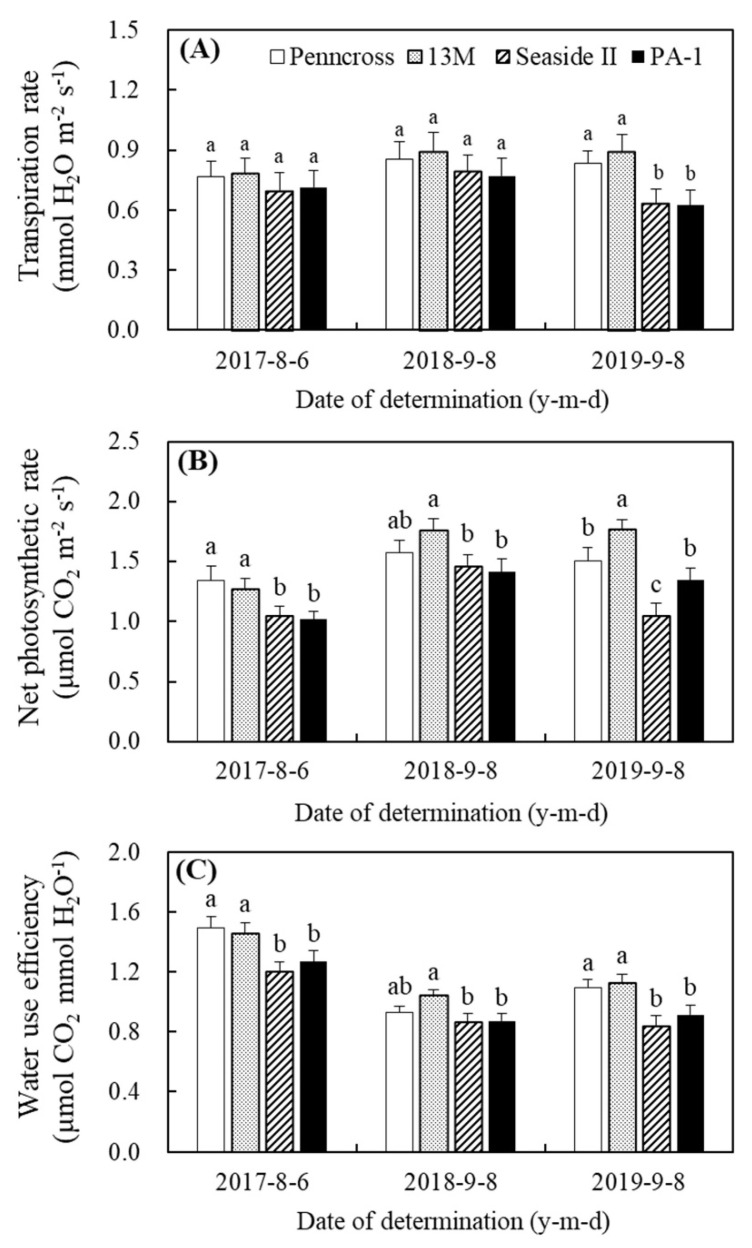
Change in (**A**) transpiration rate (Tr), (**B**) net photosynthetic rate (Pn), and (**C**) water use efficiency (WUE) of four different creeping bentgrass cultivars (Penncross, 13M, Seaside II, and PA-1) during summer in (**A**) 2017, (**B**) 2018, and (**C**) 2019. Vertical bars indicate ±SE of mean (n = 4). Different letters indicate significant differences (*p* < 0.05) at a given day.

Summer stress induced a gradual decline in the Fv/Fm of the four cultivars in 2017 (Figure 4A). The decline in the Fv/Fm was at its maximum in PA-1 on 11 July 2017 (Figure 4A). Penncross and 13M had significantly higher Fv/Fm than Seaside II and PA-1 on 6 August 2017 (Figure 4A). No cultivar differences in the Fv/Fm were detected on the first two sampling dates in 2018 (9 June and 15 August) and 2019 (8 June and 8 August) (Figure 4B,C). The effects of summer stress on the PIABS were more pronounced than the Fv/Fm among the four cultivars in 2017, 2018, and 2019 (Figure 4D–F). Penncross and 13M maintained a significantly higher PIABS than Seaside II and PA-1 during the summer; however, there was no significant difference in the PIABS between Penncross and 13M or between Seaside II and PA-1 (Figure 4D–F). The Tr of the four cultivars did not show significant differences in 2017 and 2018, but Penncross and 13M maintained significantly higher Tr as compared to Seaside II and PA-1 in 2019 (Figure 5A). In 2017, Penncross and 13M exhibited a 28% increase in Pn over Seaside II and PA-1 in the summer (Figure 5B). In the summer of 2018, 13M showed the highest Pn of the four cultivars (Figure 5B). The 13M cultivar also had a 17%, 69%, and 31% significantly higher Pn than Penncross, Seaside II, or PA-1 in the summer of 2019, respectively (Figure 5B). A significantly higher WUE was detected in Penncross and 13M than in Seaside II and PA-1 in the summer of 2017 and 2019 (Figure 5C). In 2018, 13M maintained 12%, 21%, and 20% higher WUE than Penncross, Seaside II, and PA-1 in the summer, respectively (Figure 5C).

### 3.2. Water Status and Osmolytes Affected by Summer Stress

**Figure 6:**
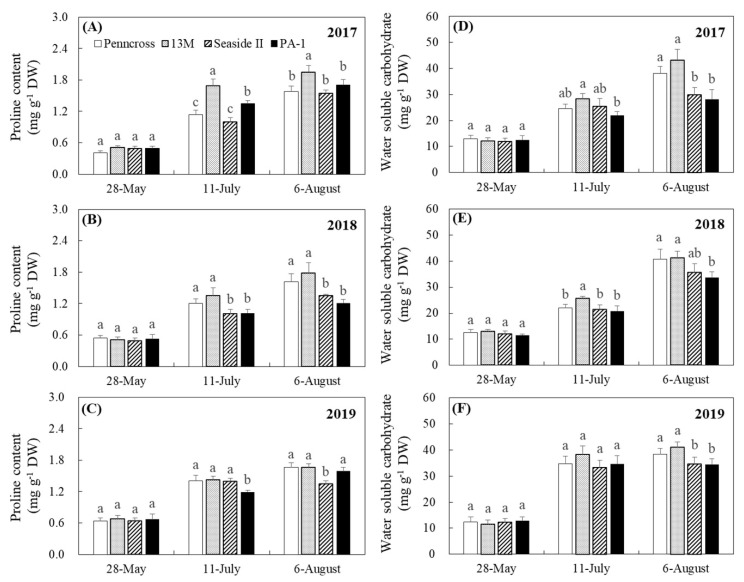
Change in (**A**–**C**) proline (Pro) content and (**D**–**F**) water-soluble carbohydrate (WUE) of four different creeping bentgrass cultivars (Penncross, 13M, Seaside II, and PA-1) during summer in (**A**) 2017, (**B**) 2018, and (**C**) 2019. Vertical bars indicate ±SE of mean (n = 4). Different letters indicate significant differences (*p* < 0.05) at a given day.

**Figure 7:**
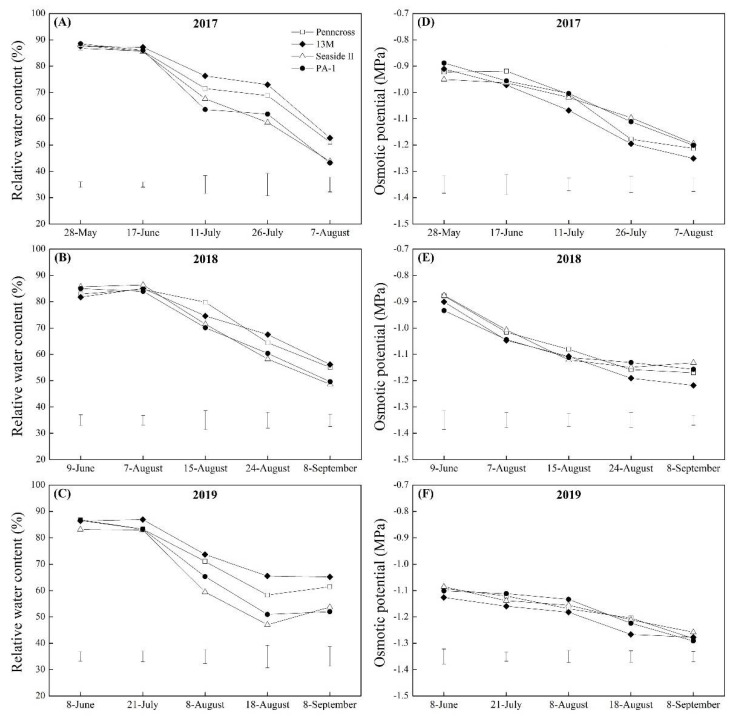
Change in (**A**–**C**) relative water content (RWC) and (**D**–**F**) osmotic potential (OP) of four different creeping bentgrass cultivars (Penncross, 13M, Seaside II, and PA-1) during summer in (**A**) 2017, (**B**) 2018, and (**C**) 2019. Vertical bars below curves indicate least significant difference (LSD) values (*p* < 0.05) at a given day.

The Pro and WSC contents in the four cultivars increased gradually from May to August in 2017, 2018, and 2019 (Figure 6A–F). There were no significant differences in Pro and WSC contents among the four cultivars in May 2017, 2018, or 2019 (Figure 6A–F). The 13M and the PA-1 cultivars had the maximum and second-highest Pro content when compared to Penncross and Seaside II in July 2017 (Figure 6A). The Pro content was significantly higher in 13M in August 2017 than in the other cultivars (Figure 6A). Significantly higher Pro content was detected in the leaves of Penncross and 13M as compared to that in the leaves of Seaside II and PA-1 in July and August of 2018 (Figure 6B). The lowest Pro content was observed in PA-1 and Seaside II in July and August 2019, respectively (Figure 6C). The 13M cultivar showed the highest WSC accumulation out of the four cultivars in July 2017, 2018, and 2019 (Figure 6D,E). In August 2017, 2018, and 2019, Penncross and 13M accumulated more WSC than Seaside II and PA-1 (Figure 6D–F). Changes in the RWC and OP showed similar trends in the four cultivars, as demonstrated by the gradual declines during summer stress in 2017, 2018, and 2019 (Figure 7A–F). The 13M cultivar maintained a higher RWC than the other three cultivars during summer stress in 2017 and 2019 (Figure 7A,C). On 8 September 2018, Penncross and 13M had a 13% increase in RWC compared to Seaside II and PA-1 (Figure 7B). OP in 13M was maintained at the lowest levels when compared to the other three cultivars in response to high temperatures during the summer in 2017, 2018, and 2019 (Figure 7D–F).

### 3.3. Oxidative Damage and Membrane Stability Affected by Summer Stress

**Figure 8:**
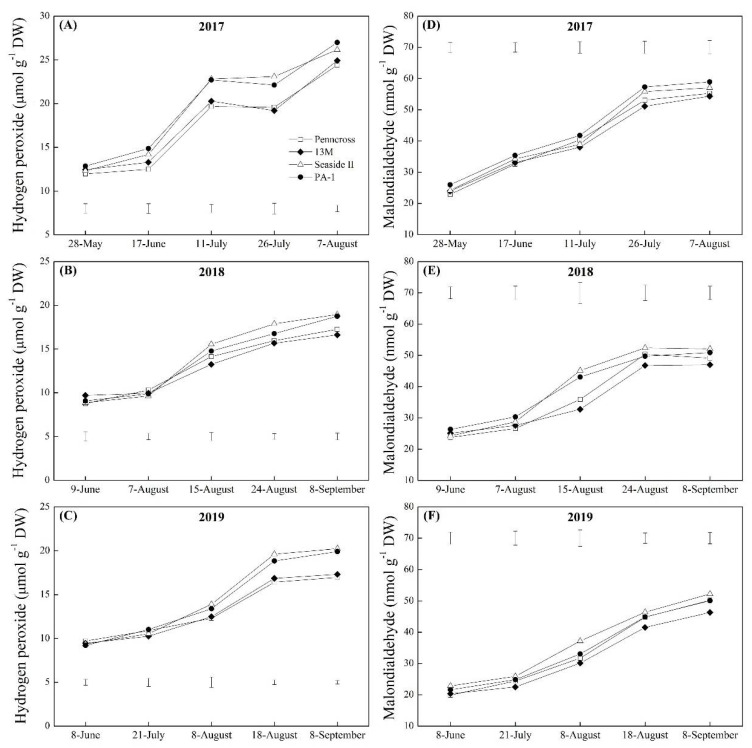
Change in (**A**–**C**) hydrogen peroxide (H_2_O_2_) content and (**D**–**F**) malondialdehyde (MDA) content of four different creeping bentgrass cultivars (Penncross, 13M, Seaside II, and PA-1) during summer in (**A**) 2017, (**B**) 2018, and (**C**) 2019. Vertical bars below or above curves indicate least significant difference (LSD) values (*p* < 0.05) at a given day.

**Figure 9:**
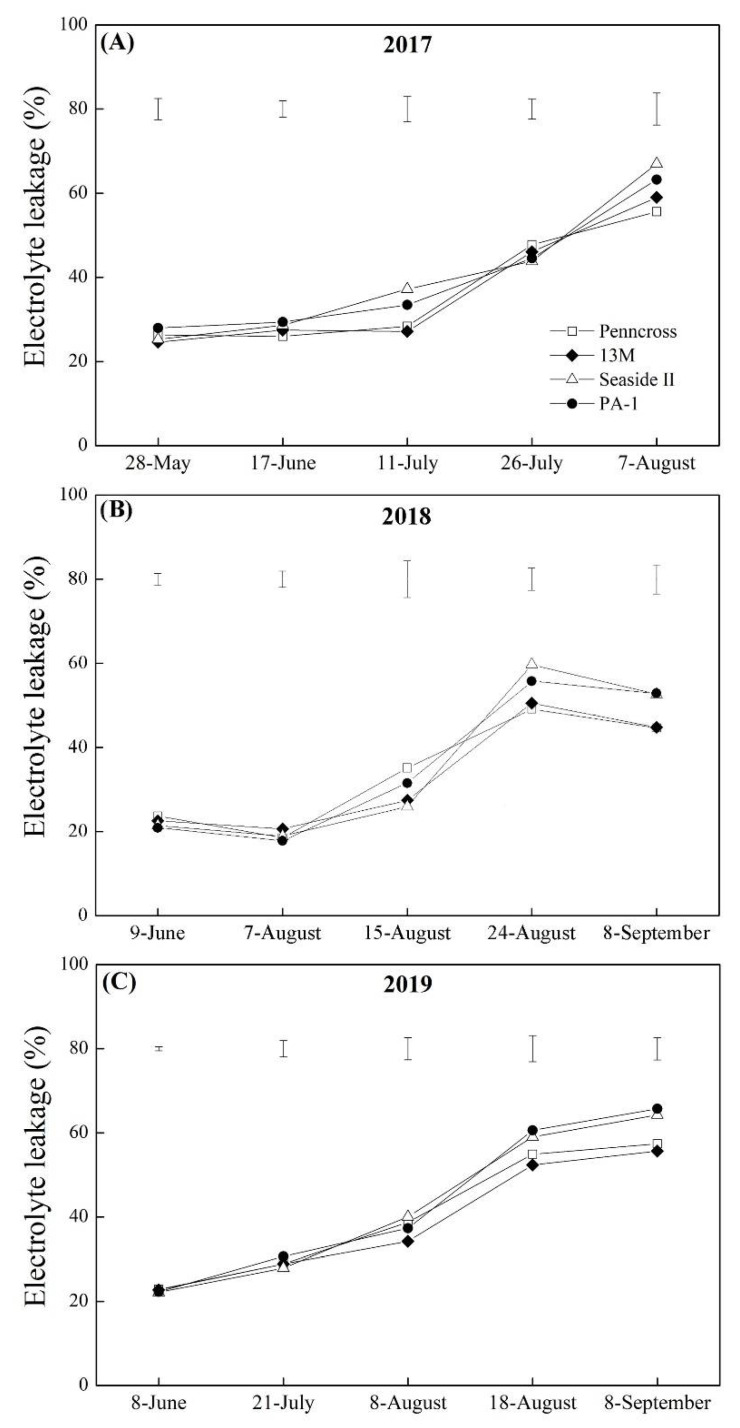
Change in electrolyte leakage (EL) of four different creeping bentgrass cultivars (Penncross, 13M, Seaside II, and PA-1) during summer in (**A**) 2017, (**B**) 2018, and (**C**) 2019. Vertical bars above curves indicate least significant difference (LSD) values (*p* < 0.05) at a given day.

The H_2_O_2_ and the MDA contents in the four cultivars increased significantly from May to September in 2017, 2018, and 2019 (Figure 8A–F). Seaside II and PA-1 accumulated significantly higher H_2_O_2_ content than Penncross and 13M from July to August in 2017 and 2018 (Figure 8A,B). The 13M cultivar maintained the lowest H_2_O_2_ content out of the cultivars during summer stress in 2018 (Figure 8B). An 18% increase in H_2_O_2_ content was detected in Seaside II and PA-1 over Penncross and 13M on August 18 and September 8, 2019 (Figure 8C). In the year 2019, PA-1 exhibited the highest MDA content of the cultivars from May to August (Figure 8D), and Seaside II and 13M had the highest and lowest MDA content of the cultivars during summer stress in 2018 and 2019, respectively (Figure 8E,F). The EL in the leaves of the four cultivars increased significantly from June to September in 2017, 2018, and 2019 (Figure 9A–C). The EL was the lowest in Penncross when compared to the other three cultivars on 7 August 2017 (Figure 9A). Seaside II and PA-1 exhibited an 18% and 16% increase in EL over Penncross and 13M on 8 September 2018 and 2019, respectively (Figure 9B,C). The 13M cultivar maintained the lowest EL level in its leaves during summer stress in 2018 and 2019 (Figure 9B,C).

## 4. Discussion

Creeping bentgrass is used as sports turfs and often needs high cultural inputs for adequate performance and functionality. The maintenance of higher TQ on golf greens during hot summer months provided better playability to golfers [6]. However, the TQ of creeping bentgrass significantly declines during the summer months when the ambient temperature exceeds its optimum growth temperature [1]. Our current study demonstrated that heat waves in the summer significantly decreased the TQ of four creeping bentgrass cultivars (Penncross, 13M, Seaside II, and PA-1) in 2017, 2018, and 2019, but 13M and Penncross could maintain a higher TQ than Seaside II and PA-1 during the summer months. A decline in TQ is characterized by a reduced grass density and yellowing leaf tissue due to accelerated leaf senescence under high-temperature stress [31,32]. Gradual declines in Chl content were observed in the four cultivars, which was consistent with the significant decreases in Fv/Fm, PIABS, and Pn. Previous studies have found that higher Chl content was a key indicator for selecting heat-tolerant genotypes in creeping bentgrass species [7,8]. Delayed Chl degradation and the higher photochemical efficiency of PSII and Pn were beneficial for better adaptation to high temperatures in creeping bentgrass during the summer months, because metabolites produced from photosynthesis provided an available energy supply for growth maintenance [13,15]. The 13M and Penncross cultivars exhibited significantly higher Chl content and photosynthesis than Seaside II and PA-1 in the summer, which could indicate that those two cultivars had better adaptability to high temperatures in the subtropical zone.

Reduced available carbohydrates limited energy supply for plant growth and development associated with heat-induced photoinhibition in plants [33]. It has been found that carbohydrate accumulation benefits creeping bentgrass against drought or heat stress [12,16,34]. An earlier study on putting green also showed that the heat-tolerant creeping bentgrass cultivar L-93 exhibited significantly higher carbohydrate contents than Penncross in response to summer heat stress in Manhattan [2]. In addition, proline accumulation and metabolism are important survival strategies against heat stress, owing to the protective functions of osmotic adjustment and ROS scavenging ability in plants [11]. High temperatures cause physiological drought mainly due to reduced water absorption in roots and accelerated transpiration in leaves. The positive effects of the accumulation of proline and carbohydrates on water homeostasis have been proved in creeping bentgrass and other plant species under high-temperature stress [35,36,37,38]. Leaf RWC and OP gradually declined with the development of summer stress, whereas proline and carbohydrates significantly accumulated in the four cultivars during the summer months. Interestingly, 13M and Penncross accumulated more proline and carbohydrates as well as a better leaf water status and osmotic adjustment ability than Seaside II and PA-1 in the summers of 2017, 2018, and 2019. These findings indicated that the cultivars’ variations in their adaptations to high temperatures could be associated with the modification of water homeostasis during the summer.

The two major indicators of membrane peroxidation were H_2_O_2_ and MDA accumulation, and their accumulations with high levels of toxicity to cells accelerated senescence under heat stress [39]. In addition, the EL level was inversely correlated to heat tolerance in creeping bentgrass species [7]. Persistent high-temperature stress induced gradual increases in H_2_O_2_ content, MDA accumulation, and EL levels in the four creeping bentgrass cultivars from June to August in 2017, 2018, and 2019. These findings indicated that the four cultivars suffered from serious oxidative damage to their cell membranes during the summer months. However, 13M and Penncross could maintain lower EL, H_2_O_2_, and MDA than Seaside II and PA-1 in the summer. Similar results were found in a previous study, which demonstrated that a lower lipid membrane peroxidation level was beneficial to the alleviation of SBD [18]. The better maintenance of cell membrane stability was also propitious to photosynthesis and metabolic activity in plants under high-temperature environmental conditions [40]. Proline accumulation and metabolism have been known to confer heat tolerance in plants associated with ROS detoxifying and delayed senescence [38,41]. An exogenous application of proline could also mitigate the detrimental effects of high temperatures on creeping bentgrass in relation to delayed leaf senescence [15].

## 5. Conclusions

Summer stress significantly decreased the TQ of four creeping bentgrass cultivars (13M, Penncross, Seaside II, and PA-1) in 2017, 2018, and 2019. A variety of physiological processes were affected by heat stress in the summer, including significant declines in Chl content, photosynthesis, leaf RWC, and OP as well as obvious increases in carbohydrates, proline, H_2_O_2_, MDA, and EL in the four cultivars. The 13M and Penncross cultivars exhibited better performance than Seaside II and PA-1 in response to summer stress from 2017 to 2019, which was associated with the maintenance of better photosynthesis, water status, osmolytes accumulation, and cell membrane stability. The 13M and Penncross cultivars could be used as potential candidates for turf establishment in a subtropical zone. An in-depth understanding of physiological responses to summer stress also provided critical information for the breeding and development of germplasm with heat tolerance in creeping bentgrass species.

## Data Availability

All data presented in this study are available in the article.
